# Potent Antifungal Properties of Dimeric Acylphloroglucinols from *Hypericum mexicanum* and Mechanism of Action of a Highly Active 3′Prenyl Uliginosin B

**DOI:** 10.3390/metabo10110459

**Published:** 2020-11-13

**Authors:** Noemi Tocci, Tobias Weil, Daniele Perenzoni, Marco Moretto, Nicolai Nürk, Santiago Madriñán, Ruggero Ferrazza, Graziano Guella, Fulvio Mattivi

**Affiliations:** 1 Research and Innovation Centre, Fondazione Edmund Mach, 38010 San Michele all’Adige (TN), Italy; noemi.tocci26@gmail.com (N.T.); tobias.weil@fmach.it (T.W.); daniele.perenzoni@fmach.it (D.P.); marco.moretto@fmach.it (M.M.); 2 Department of Plant Systematics, BayCEER, University of Bayreuth, 95447 Bayreuth, Germany; nuerk@uni-bayreuth.de; 3 Departamento de Ciencias Biológicas, Universidad de los Andes, Bogotá 111711, Colombia; samadrin@uniandes.edu.co; 4 Jardín Botánico de Cartagena “Guillermo Piñeres”, Turbaco, Bolívar 131007, Colombia; 5 Department of Physics, University of Trento, 38123 Trento, Italy; r.ferrazza@unitn.it; 6 Department of Cellular, Computational and Integrative Biology, CIBIO, University of Trento, 38122 Trento, Italy

**Keywords:** *Hypericum*, bioactive compounds, antifungal activity, acylphloroglucinols, structural annotation, nuclear magnetic resonance spectroscopy, mass spectrometry, cytotoxicity, mechanism of action

## Abstract

The success of antifungal therapies is often hindered by the limited number of available drugs. To close the gap in the antifungal pipeline, the search of novel leads is of primary importance, and here the exploration of neglected plants has great promise for the discovery of new principles. Through bioassay-guided isolation, uliginosin B and five new dimeric acylphloroglucinols (uliginosins C-D, and 3′prenyl uliginosins B-D), besides cembrenoids, have been isolated from the lipophilic extract of *Hypericum mexicanum*. Their structures were elucidated by a combination of Liquid Chromatography - Mass Spectrometry LC-MS and Nuclear Magnetic Resonance (NMR) measurements. The compounds showed strong anti-*Candida* activity, also against fluconazole-resistant strains, with fungal growth inhibition properties at concentrations ranging from 3 to 32 µM, and reduced or absent cytotoxicity against human cell lines. A chemogenomic screen of 3′prenyl uliginosin B revealed target genes that are important for cell cycle regulation and cytoskeleton assembly in fungi. Taken together, our study suggests dimeric acylphloroglucinols as potential candidates for the development of alternative antifungal therapies.

## 1. Introduction

Fungal infections are an increasingly serious problem especially in patients with immune deficiencies caused by diseases like human immunodeficiency virus / acquired immunodeficiency syndrome (HIV/AIDS) and cancer or by prolonged antibiotic treatments [1]. In particular, superficial or invasive infections and chronic conditions caused by fungi annually affect millions of people, leading to gigantic treatment costs and a death toll of more than one million people per year [2]. Species belonging to the genera *Candida* are among the most prevalent cause of invasive infections such as candidiasis and candidemia [3,4]. The treatment outcomes of *Candida* infections are often impaired due to the low availability of therapeutic options that are mainly restricted to the four antifungal classes azoles, polyenes, echinocandins, and pyrimidine analogs [5]. Importantly, the success of the treatments is threatened by the increasing emergence of multidrug-resistant pathogens, especially for the *Candida* species *C. glabrata, C. parapsilosis, C. lusitaniae*, and *C. tropicalis*, with the estimation that more than 20–30% of candidemia cases involve fungal species with intrinsic resistance to either fluconazole or echinocandins [6]. Overall, the outcomes of the available antifungal therapy are not satisfactory, and therefore the discovery of new active compounds is a priority considering this alarming gap in the anti-fungal pipeline [7]. The search for novel antifungal agents from medicinal plant sources [8,9] offers a promising path to addressing this gap.

Among the natural compounds, dimeric phloroglucinols have recently attracted attention, mainly because of their high degree of chemical diversity and their promising activity as cytotoxic and antimicrobial drugs, which make these compounds a fascinating target for pharmacological research [10].

Dimeric phloroglucinols are not universally biosynthesized in plants but mainly limited to a small number of genera [10]. The plant genus *Hypericum,* including many medicinal species, is considered as a prolific source of phloroglucinols. Despite the genus being mainly known for the extensively investigated *H. perforatum*, an increasing number of publications is calling attention towards less distributed or neglected species as well for the discovery of new antifungal formulations and compounds [8,9,10]. Here, in particular, lipophilic extracts obtained from *Hypericum* species in South America have shown inhibitory activity on the growth of human fungal pathogens [8,9,10] and their properties have been suggested to be related to the presence of a class of dimeric phloroglucinols: the dimeric acylphloglucinols (DAPs) [11] consisting of filicinic acid and phloroglucinol moieties [12] Despite available studies documenting a great potential for this class of compounds as antimicrobial, antidepressant and cytotoxic drug leads, DAPs are poorly investigated, maybe due to difficulties in isolation, low yield, and instability [13].

In the search for new antifungal leads, we investigated the chemistry and the antifungal properties of extracts from *Hypericum mexicanum* L., a neglected species [14] that is part of a recent and rapid radiation in the montane páramos of the Colombian and Venezuelan Andes [12,15] where it is a common weed and used by the native populations for the manufacture of handicraft products, and remedies for the treatment of wounds and burns (aerial parts) and muscle aches and kidney disease (roots) [16]. However, literature on medicinal applications of *H. mexicanum* extracts is very scarce. Corso-Barragan et al. (2017) [17], reported the antibacterial activity of the ethanolic extract of the plant, while essential oils of *H. mexicanum* have been suggested as potential repellents in plant pest control [18].

Our study leads to the description of the phenolic profile of the whole plant, and to the isolation and characterization of five novel DAPs that showed selective inhibitory properties on fungal growth and no cytotoxicity on human cell lines. Notably, the first evidence for the mechanism of action of a highly active DAP, a sarothralen B-like compound, is reported as deduced from the output of a chemogenomic screening.

## 2. Results and Discussion

### 2.1. Chemical Profile of Hypericum Mexicanum

Crude extracts obtained from leaves, stems and roots of *H. mexicanum* were chemically characterized by the identification and quantification of 34 low molecular weight polyphenols as reported in Table 1. Five metabolites (3,5-dihydroxy benzoic acid, quercetin-3-glucoside, procyanidin B2, kaempferol-3-glucoside and phlorizin) were distributed in all of the organs, while three only in the aerial parts, and five compounds detected in both leaves and roots but not in stems. Leaves extract was the richest in phenols and the more abundant compounds were procyanidin B2 (117.37 ± 4.02 µg/g); quercetin (97.07 ± 1.01 µg/g); isorhamnetin-3-glucoside (96.58 ± 5.01 µg/g); quercetin-3-glucoside (86.45 ± 3.03 µg/g); and epicatechin (36.17 ± 2.02 µg/g). The chemical profile of *H. mexicanum* resembles those of the closely phylogenetically related species *H. laricifolium* [8], strengthening our previous observation that chemical profiles in *Hypericum* seem to mirror evolutionary relationships of species.

### 2.2. Antifungal Activity of H. Mexicanum Extracts and Chemical Analysis of the Chloroformic Extract

The extract from the aerial parts was tested against two environmental strains of *S. cerevisiae* and 3 clinical strains of *C. albicans* showing encouraging inhibition properties at relative low concentration (44.70 µg/mL for *S. cerevisiae* and 63.50 µg/mL for *C. albicans*) (Table S1). To better understand the nature of the active principles present in the crude extracts, leaves of *H. mexicanum* were additionally subjected to extraction with chloroform, obtaining an extract enriched in molecules with different polarity that was tested against a broader panel of *Candida* spp. clinical isolates (Table 1).

The chloroform extract showed the strongest antifungal activity against all tested strains when compared with the methanolic extract (Table 2 and Table S2).

Chemical analysis showed low amounts of phenols in comparison with the methanolic extract (Table 1), suggesting that the observed antifungal properties could be attributed to the presence of other compounds with more lipophilic characteristics in line with what was found for other *Hypericum* species phylogenetically close to *H. mexicanum* [9,19,20].

### 2.3. Bioassay-Guided Isolation and Characterization of Antifungal Principles

To better define the antifungal properties of the lipophilic extracts, the aerial parts of *H. mexicanum* were extracted with hexane and subsequently partitioned with *n*-hexane and water. The *n*-hexane soluble compounds were separated by silica-gel column chromatography obtaining two major fractions:

Fraction I appeared to be mainly composed of a single species according to HPLC chromatogram and UV spectrum (Figure S1). NMR analysis of fraction I confirmed the presence of a main terpenoid compound (93%), cembrene, and of a minor terpenoid compound (7%), isocembrene (data not shown).

Fraction II was composed of six compounds as revealed by HPLC chromatogram and UV spectrum (Figure S2). The anti-fungal test outlined the fraction II as the most active in the inhibition of *Candida* growth as showed in Table 3).

**Figure 1:**
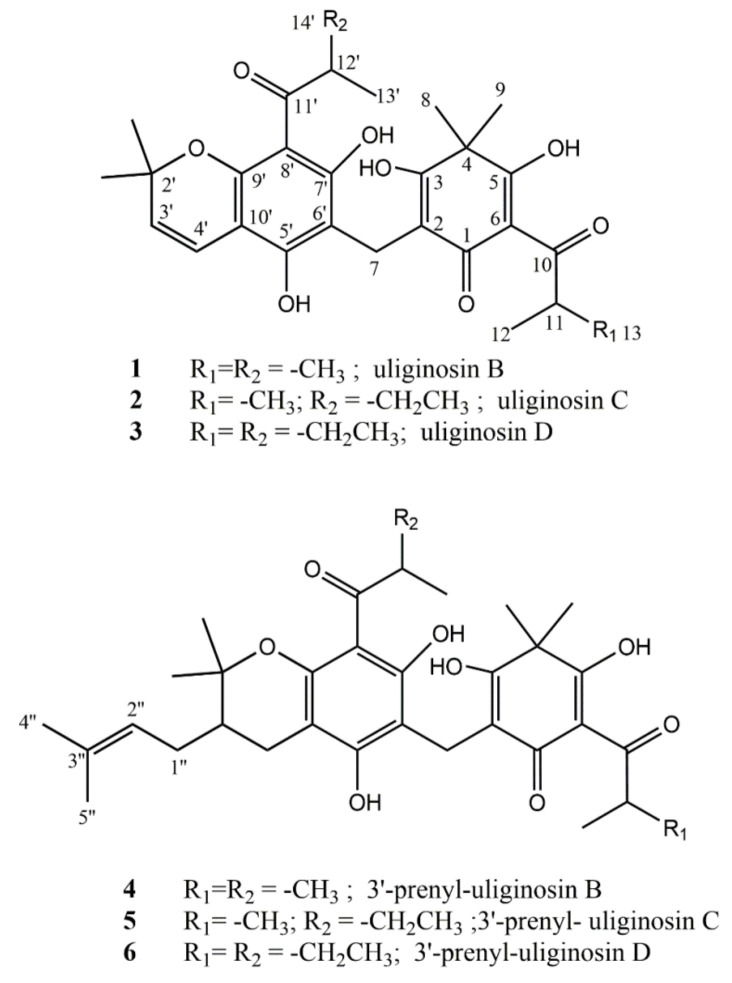
Structural elucidation.

Among the latter, we isolated after extensive chromatographic purification both the known dimeric acylphloroglucinols uliginosin B (**1**) and its new analogues uliginosin C (**2**), uliginosin D (**3**) where the 2-methyl-1-oxopropyl moiety of **1** are replaced by the 2-methyl-1-oxobutyl moiety at C-8′ in **2** and both at C-6 and C-8′ in **3**. We also isolated the corresponding 3′ prenylated dimeric acylphloroglucinols analogues of **1-3** which we named 3′prenyl uliginosin B (**4**), 3′prenyl uliginosin C (**5**) and 3′prenyl uliginosin D (**6**). Compounds structures are reported in Figure 1.

The structural elucidation of uliginosin C (**2**) was straightforward due to the strong structural resemblance with the well-known uliginosin B [13], also present in our extract.

In particular, high resolution mass measurements carried out by ESI in negative ion mode (HR-ESI-MS) of **2** showed a pseudomolecular ion peak [M − H]^−^ at *m*/*z* 511.2315 ± 0.0010, consistent with a molecular formula of C_29_H_35_O_8_ (calculated exact mass 511.2337) for the anionic form of **2**, thus speaking for the presence of one methylene (–CH_2_–) more in **2** with respect to **1**. Extensive 2D-NMR measurements on **2** allowed us to establish that this methylene unit must be part of one ethyl moiety at C-11′ replacing the methyl analogue in **1,** or in more general terms, we established that **2** is the 2-methyl-1-oxobutyl analogue of **1**. Whereas from one side the presence of this moiety could be easily inferred by the analysis of the 1D-NMR spectrum (the coupling pattern of the secondary proton as expected was found as a sextet), from the other side Heteronuclear Multiple Bond Correlation (HMBC) correlations indicated that the 2-methyl-1-oxobutyl moiety was linked to C-11′, leaving the remaining 2-methyl-1-oxopropyl unit linked to C-10. In particular, the proton at δ_H_ 3.83 with a sextet pattern (J = 6.7 Hz) was found to be ^1^J hetero-correlated (Heteronuclear Single Quantum Coherence, HSQC) with ^13^C resonance at δ_C_ 45.7; more important, in our HMBC measurements it was found to be strongly (^3^J) long-range hetero-correlated to δ_C_ 11.9 (methyl carbon of the ethyl moiety) and to δ_C_ 104.2 (easily assigned to C-8′) and to show weak (^2^J) hetero-correlations with δ_C_ 26.7 (attributable to C-14′) and with δ_C_ 210.8 (C-11′ carbonyl group). A full ^1^H and ^13^ C-NMR assignment of **2** is reported in Table 4. In order to confirm assignments made in CDCl_3_, further NMR experiments of 1 were also recorded in acetone-d6 (not reported).

**Figure 2:**
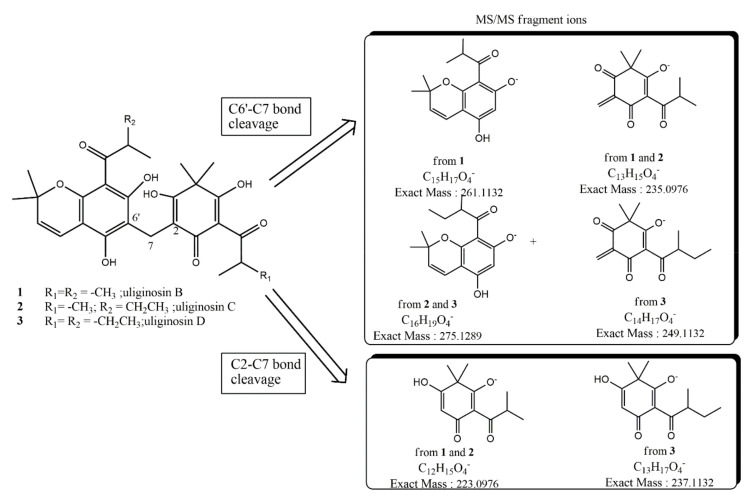
Observed ion fragments upon MS-MS fragmentation of the parent [M-H]- of uliginosins C (2) and D (3).

The structure is also fully supported by tandem ESI MS experiments that showed characteristic fragments ions at *m*/*z* 235 and 275 following the breaking of the C6′-C7 bond and the fragment ion at *m*/*z* 223 following the breaking of the C2–C7 bond. When we compare the results of similar MS/MS fragmentation of uliginosin B (**1**) leading to fragment radical ions at *m*/*z* 235 and 261 (via C6′–C7 homolytic bond breaking) and fragment ion at m/z 223 (via C2–C7 bond breaking), our results in **2** are only compatible with a structure where the 2-methyl-1-oxobutyl unit is installed at C-8′, as drawn in Figure 2.

High-Resolution Quadrupole-Time of Flight Mass Spectrometry with Electrospray Ionization on a (HR-ESI-QTOFMS) of compound **3** showed a pseudomolecular ion peak [M − H]^−^ at *m*/*z* 525.2483 ± 0.0010, consistent with a molecular formula of C_30_H_37_O_8_ (calculated exact mass 525.2493) for the anionic form of **3,** thus speaking for the presence of two methylenes (–CH_2_–), more in **3** with respect to **1**; our guess of the presence in **3** of the 2-methyl-1-oxobutyl moiety both at C-6 and C-11′ was promptly confirmed by the results of tandem ESI MS (Figure 2) where the characteristic fragment radical ions were detected at *m*/*z* 249 and 275 (via C6′–C7 bond breaking) and at *m*/*z* 237 (via C2–C7 bond breaking). Although **3** was isolated only in a low amount preventing extensive NMR measurements, its 1H-NMR spectrum was in full agreement with the proposed structure showing two characteristic multiplets at δ_H_ 3.82 (H-12′, sextet J = 6.7) and at δ_H_ 4.06 (H-11, sextet J = 6.7). Difficulties in NMR interpretation mainly derive from the expected keto−enol tautomerization of the cyclohexenone moiety in organic solvent solutions, a process that significantly complicates the NMR spectra of these metabolites, often hindering a complete assignment of the signals. As already observed for uliginosin B, even after extensive reverse phase HPLC purifications, in our hands these dimeric acylphloroglucinols are accompanied by a significant relative amount (20–25%) of their iso-analogues deriving from the closure of isoprenyl chain at C-10′ not at the oxygen atom at C-9′ but, instead at the oxygen atom at C-5′. As previously reported in iso-uliginosin B [13], in the ^1^H-NMR spectra of **2** and **3** besides major resonance attributable to the olefinic proton signals at C-3′ and C-4′, small resonances at slightly deshielded (+0.025 ppm for H–C3′) or slightly shielded chemical shift (−0.035 ppm for H–C-4′) also appear whilst all the other resonances of these iso-analogues are almost superimposable to the major uliginosin C (**2**) and uliginosin D (**3**).

**Figure 3:**
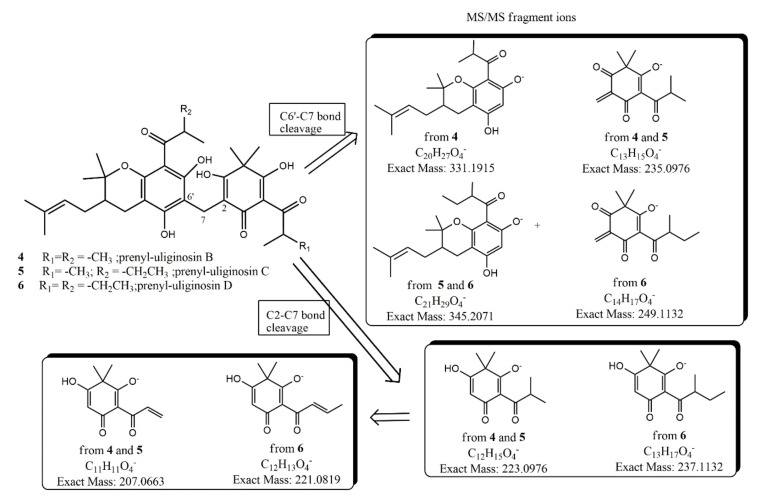
Observed ion fragments upon MS-MS fragmentation of the parent [M − H]^−^ of prenyl-uliginosins B(4), C(5), and D(6).

HR-ESI-MS of **4** showed a pseudomolecular ion peak [M − H]^−^ at *m*/*z* 567.2941 ± 0.0010, consistent with a molecular formula of C_33_H_43_O_8_ [calculated exact mass 567.2943] for the anionic form of **4,** thus containing an isoprenyl chain (C_5_H_10_) more than uliginosin B (1). In the 1H-NMR spectrum of **4**, the most prominent feature was the absence of the olefinic protons at C-3′ and C-4′ of uliginosin B-D. After extensive 2D NMR measurements, we unambiguously establish that this prenyl chain was linked to C-3′, leaving C-4′ as a saturated sp^3^ carbon atom, as drawn in Figure 3.

This outcome was also in agreement with tandem Mass Spectrometry with Electrospray Ionization (ESI-MS/MS) measurements showing fragment radical ions deriving from the C6′–C7 bond breaking at *m*/*z* 235 and 331 and from the C2–C7 bond breaking at *m*/*z* 223 (Figure 3). The key ^1^H-^13^C NMR hetero-correlations are those deriving from long range couplings of C-2′ (singlet at δ_C_ 79.5) both with 3H-C-2′ (broad singlets at _δH_ 1.47 and 1.23) and with diastereotopic protons at C-4′ (δ_H_ 2.83 dd, 5.5, 17.4; δ_H_ 2.17 dd, 10.3, 17.2). On the other hand, strong couplings with the same protons were also found with C-3′ (doublet at δ_C_ 40.8). Since in the HSQC spectrum C-3′ was found carbon-coupled to a broad multiplet at δ_H_ 1.71, which, in turn, in the COSY experiment was found proton-coupled to methylene geminal protons both at C-4′ and C-1” (broad multiplets at δ_H_ 2.32 and 1.81), the position of the targeted isoprenyl chain must be at C-3′ (equatorial position) of the dihydropyran ring. Although the structure of **4** has recently been reported and named denudatin A [21], two critical issues emerge from this report. First of all, some NMR assignments are inconsistent and incorrect attributed. In fact, (i) allylic protons at C-1” reported there as br singlet at δ_H_ 1.48 must be assigned to the diastereotopic signals at δ_H_ 2.32 and 1.81, being ^3^J homonuclear coupled to the olefinic proton of the prenyl chain; (ii) the geminal methyls at C-2′ (quartets δ_C_ 27.5/δ_H_ 1.47 and δ_C_ 20.5/δ_H_ 1.23) show very similar long range couplings to C-2′ and C4′. Second, and most important, the name denudatin A should not have been used for this compound, since it had already been given several years ago (1980) to another isolated natural compound isolated from *Magnolia denudata* Desr [22]. Surprisingly enough, a structure-based search of **4** in the database Scifinder [23] does not lead to any literature reference related to acylphloroglucinols, including reference [21]; in contrast, searching the same database with the name-based keyword “denudatin A” revealed 17 literature references, of which just one [21] was related to this prenylated acylphloroglucinol **4**. However, and really noteworthy, the CAS Registry Number (87402-87-7) assigned to **4** is wrong, being the same as that assigned to the previously reported neolignan metabolite from *M. denudata*. The structural elucidation of prenyl-uliginosin C (**5**) was straightforward due to the strong structural resemblance with its analogue **4**. In particular, HR-ESI-MS of **5** (Table 5) showed a pseudomolecular ion peak [M − H]^−^ at *m*/*z* 581.3111 ± 0.0010, consistent with a molecular formula of C_34_H_45_O_8_ (calculated exact mass 581.3120) for the anionic form of **5,** thus indicating the presence of one methylene (-CH_2_-) more in **5** with respect to **4**. 1D and 2D-NMR spectra of **5** allowed us to establish that this methylene unit must be part of one ethyl moiety at C-11′, fixing **5** as the 2-methyl-1-oxobutyl analogue of **4,** in full agreement with the results of tandem-MS measurements (Figure 3). HR-ESI-MS (Table 5) of prenyl-uliginosin B (**6**) showed a pseudomolecular ion peak [M − H]^−^ at *m*/*z* 595.3267 ± 0.0010, consistent with a molecular formula of C_35_H_47_O_8_ [calculated exact mass 595.3276] for the anionic form of **6,** thus bearing two methylenes (–CH_2_–) more than prenyl-uliginosin B (**4**)**.** On the other hand, the presence in **6** of the 2-methyl-1-oxobutyl moiety both at C-6 and C-11′ was fully supported by the results of tandem ESI-MS (Figure 3), where the characteristic fragment radical ions were detected at *m*/*z* 249 and 345 (via C6′–C7 bond breaking) and at *m*/*z* 237 (via C2–C7 bond breaking). Although **6** was isolated in a very low amount and pure enough for extensive NMR measurements, the ^1^H-NMR spectrum was in full agreement with the proposed structure showing two characteristic multiplets at δ_H_ 3.82 (H-12′, sextet J =6.7) and at δ_H_ 4.06 (H-11, sextet J =6.7).

### 2.4. Antifungal and Cytotoxic Properties of Isolated Compunds

The inhibitory activity of the isolated compounds was tested against clinical isolates of *Candida* strains, and their cytotoxicity was evaluated against the human skin fibroblast Hs27 cell line (*ATCC* ^®^ CRL-1634™, Manasas, VA, USA) and against peripheral blood mononucleate cells (PBMC). All isolated compounds showed strong antifungal activity against *Candida* species at concentrations ranging from 3 to 32 µM (Table S3). The uliginosin B-like and sarothralen B-like compounds 2 and 4 were selected for in-depth biological analysis. Both compounds showed a potent antifungal activity against a broad panel of *Candida* species and, in particular, they out-competed the main antifungal fluconazole against azole-resistant *C. albicans* and *C. parapsilosis* (Table 6).

**Figure 4:**
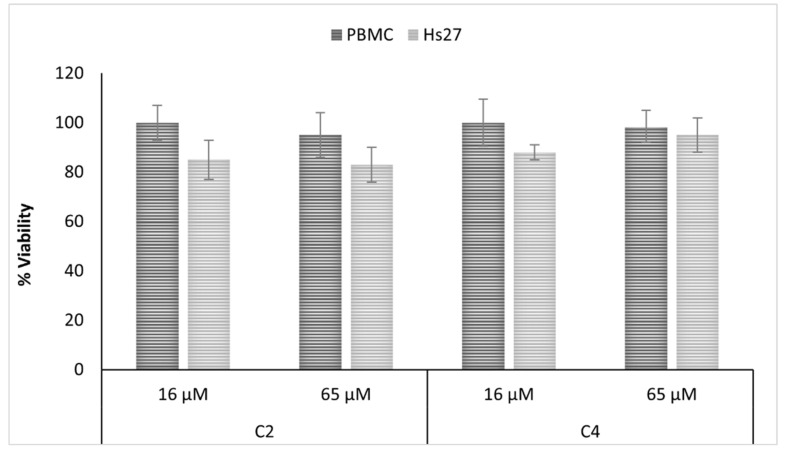
Evaluation of the cytotoxicity of uliginosin C (C2) and 3′prenyl uliginosin B (C4), measured using the WST-8 conversion assay, on normal human cell lines. The metabolic activity is given as the endpoint of toxicity in human peripheral mononucleate cells (PBMC) and human skin fibroblast cell line (Hs27), following 24 h exposure to the compounds. Values represent standard error of the mean (*n* = 3). The results were analyzed by Student’s *t*-test. No statistically significant differences were detected.

Additionally, both compounds showed no or only reduced cytotoxicity when tested against normal human cell lines at concentrations that were twice as high as the highest measured antifungal activity (Figure 4).

### 2.5. Mechanism of Action Analysis

**Figure 5:**
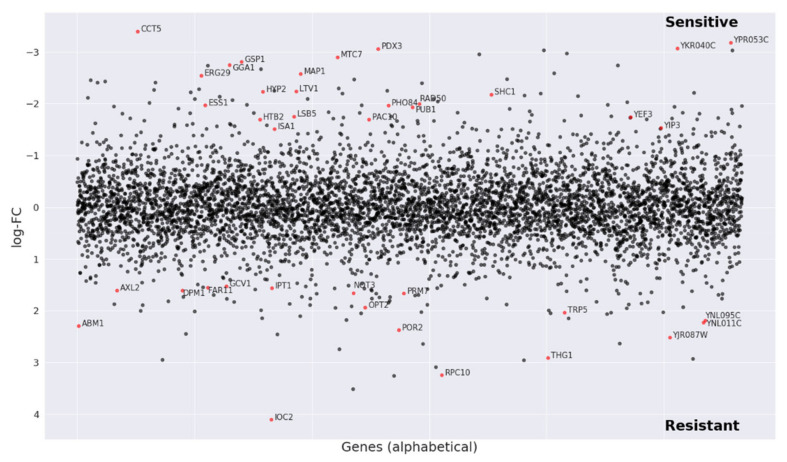
Chemogenomic screen. The pool of tagged 5936 deletion mutants was grown for 20 generations in the presence of 3′prenyl uliginosin B (150 µg) and 1% DMSO (control). Log2 ratio (control intensity/treatment intensity) was calculated and plotted as a function of gene. The genome-wide readout of heterozygous highly sensitive strains included CCT5, a subunit of the cytosolic chaperonin CCT ring complex that is required for the assembly of actin and tubulins (red dots: logFC > 1.5 and *p*-value < 0.01).

As sarothralen B-like compounds have not yet been described as antifungals, we subjected 3′prenyl uliginosin B to a chemogenomic screen to identify possible molecular targets (Figure 5).

Chemogenomic profiling allows characterizing the global cellular response to a compound of interest and to identify all gene products that functionally interact with this compound by using a genome-wide competitive growth assay [24]. Here, each member of a molecularly bar-coded pooled yeast deletion collection is grown in a single culture, in either the presence or absence of 3′ prenyl uliginosin B, thus allowing one to record their growth fitness in parallel. Hence, deletion strains having a reduced fitness when compared to the control indicate that the respective gene deletion grants sensitivity to the compound. This chemogenomic screen revealed deletion strains highly sensitive against 3′ prenyl uliginosin B that were heterozygous for genes involved in cell cycle progression, translation, protein folding and maturation, sterol biosynthesis and uptake, telomere and chromosome maintenance, and oxidative stress resistance (Table S4). The most sensitive mutant was heterozygous for the chaperonin CCT5, a subunit of the eukaryotic cytosolic chaperonin containing TCP-1 (CCT). CCT is a multi-subunit protein complex that assists the folding of several proteins important for cell cycle regulation, cytoskeleton assembly, and chromatin remodeling [25,26]. It also plays a role in the formation of stress granulates [27] and assists the proper assembly and function of the septin ring [26]. All CCT genes are essential in yeast and small perturbations can lead to serious growth defects or loss of viability [28].

Notably, the interactions between CCT and its major folding substrates actin and tubulin seem to be sequence-specific [29], which would make it an exciting novel drug target, especially due to the evidence that changes in expression of a CCT subunit can block hyphal morphogenesis in *Candida* [30]. Hyphal growth is regarded as an important virulence factor in *Candida* species and inhibiting the yeast-to-hyphal switch during early stages of infection can significantly increase the survival of the host [4,31].

## 3. Materials and Methods

### 3.1. Plant Material

Plant material used in this study was collected in July 2012 in the vicinity of Bogotá in the eastern Cordillera of the Colombian Andes (loc. 4.771647, -73.874224) and was identified as species *Hypericum mexicanum* L. using the identification key provided in the monograph *Hypericum* [32]. Voucher specimens have been deposited in the herbarium of the Universidad de los Andes (ANDES; coll. Nürk, Dreer & Rojas Colmenares 934, det. N.M.Nürk), Colombia.

### 3.2. Preparation of Plant Extracts

For the investigation of the phenolic profile of *Hypericum mexicanum* (Table 1), roots, leaves, and stems have been left to air-dry till reaching constant weight. Dried plant biomass (10 g per each sample) was powdered by grinding and extracted with methanol (drug/solvent ratio = 1:20 *w*/*v*) by maceration (3 × 24 h) in the dark.

After extraction with methanol, a batch of dried leaves biomass was subjected to further extraction with chloroform (drug/solvent ratio = 1:20 *w*/*v*) in order to obtain an extract characterized by non-polar compounds to be tested for antimicrobial activity.

For compound isolation, dried *H. mexicanum* leaves and stems (500 g) were powdered by grinding and extracted with hexane (drug/solvent ratio = 1:20 *w*/*v*) by maceration (3 × 24 h) in the dark. The hexane extract was further extracted with 70% acetonitrile in acid water (doubly distilled water added with 0.1% formic acid). The obtained extracts were evaporated to dryness, weighed, and stored at −20 °C until further use. Extracts were resuspended in 50% methanol/water and filtered with sterile (0.2 μm) PTFE filters, prior to analysis.

### 3.3. Chemicals

Methanol and acetonitrile were of LC-MS grade and were purchased from Sigma-Aldrich (St. Louis, MO, USA). Milli-Q water was used for the chromatography. The majority of the chemical standards are commercially available and were obtained from different suppliers [33].

Cis-piceid was produced by photochemical isomerization of the trans form, as described by Mattivi et al. (1995) [34].

### 3.4. LC-MS Analysis

Analysis of phenolic metabolites was performed as previously described [33] with few modifications. A Waters Acquity UPLC system (Milford, MA, USA) consisting of a binary pump, an online vacuum degasser, an autosampler, and a column compartment was used. Phenolic compounds were separated at a temperature of 40 °C on a Waters Acquity HSS T3 column 1.8 μm, 150 mm × 2.1 mm (Milford, MA, USA). The mobile phase was composed of component A (0.1% formic acid in water) and component B (0.1% formic acid in acetonitrile). The flow was set to 0.4 mL/min, and the gradient profile was: 0 min, 5% B; from 0 to 3 min, linear gradient to 20% B; from 3 to 4.3 min, isocratic 20% B; from 4.3 to 9 min, linear gradient to 45% B; from 9 to 11 min, linear gradient to 100% B; from 11 to 13 min, wash at 100% B; from 13.01 to 15 min, then re-equilibrated to the initial conditions of 5% B. The injection volume was 2 μL for both sample and standard solutions. Each sample was analyzed in triplicate. After each injection, the needle was rinsed with 600 μL of a weak washing solution (water/methanol, 90:10) and 200 μL of a strong washing solution (methanol/water, 90:10). Samples were kept at 6 °C during the analysis.

Mass spectrometry detection was performed on a Waters Xevo TQMS (Milford, MA, USA) instrument equipped with an electrospray (ESI) source. Capillary voltage was 3.5 kV in positive mode and −2.5 kV in negative mode; the source was kept at 150 °C; the desolvation temperature was 500 °C; cone gas flow, 50 L/h; and desolvation gas flow, 800 L/h. Further MS parameters are reported in Vrhovsek et al. (2012) [33].

Quantification was done using Waters MassLynx 4.1 and TargetLynx software (Waters, Milford, MA, USA). All compounds were quantified via external calibrations.

### 3.5. Compound Isolation and Mass Spectrometric Conditions

The hexane extract was subjected to a solid phase extraction SPE (direct phase, SI), using hexane/ethyl acetate as mobile phase in a gradient elution (starting with 100% hexane and reaching up to 1:1 hexane/ethyl acetate). Several fractions were collected and spotted into a TLC plate (TLC Silica Gel 60 T_254_, from Merck, Germany); they were chromatographically resolved with a mobile phase consisting of hexane/ethyl acetate 9:1, and the analytes were visualized by carbonization with an aqueous solution of 10% cerium (IV) sulfate and 15% H_2_SO_4_. Alike fractions were grouped together, obtaining two major fractions, and the collected pooled fractions were subjected to further analysis, including NMR and LC-MS. The LC-MS measurements were carried out using a High Performance LC system (CBM-20 A, equipped with the binary pump LC-20AB, Shimadzu, Milano, Italy) working in reversed phase with a Kinetex C18 column (100 Å pore size, 4.6 mm ID, 2.6 μm particle size, and 10 cm length, Phenomenex, Bologna, Italy). The mobile phase was composed of solvent A, methanol:water (7:3 *v*/*v*) with 0.5% formic acid, and solvent B, methanol with 0.5% formic acid. The gradient elution program was tailored to each of the fractions investigated:

- Fraction I: Isocratic elution, 70% B and 30% A, flux: 0.9 mL/min;
- Fraction II: Gradient elution, starting with 75% B and reaching 85% B in 20 min.

These compositions were maintained until 30 min. The flux was set to 1.1 mL/min. The effluent from the column was split in two, and simultaneously detected by both an UV/VIS spectrometer (Shimadzu, Milano, Italy) and by an API 3000 QQQ mass spectrometer equipped with an electrospray ion source (ESI) (AB Sciex, New York, NY, USA). Both positive and negative ion scans were acquired, and the mass spectrometer was operated in full scan mode.

Isolated compounds were analyzed by Waters Acquity UPLC coupled via an electrospray ionization (ESI) interface to a Synapt HDMS QTOF MS (Waters, Manchester, UK) operating in W-mode and controlled by MassLynx 4.1 was used. All samples were analyzed on a reversed phase (RP) ACQUITY UPLC 1.8 mm, 2.1 µm × 150 mm HSS T3 column (Waters, Milford, MA, USA) at 30 °C with gradient elution starting isocratic from 100% A (water, 0.1% formic acid) from 0 till 6 min, then increasing linearly over 56 min to 100% B (methanol, 0.1% formic acid), where it was held isocratic until 60 min, with 0.3 mL/min flow rate. Injection volume was 5 mL and the samples were kept at 4 °C throughout the analysis. Mass spectrometric data were collected in negative ESI mode over a mass range of 50 to 3000 amu with scan duration of 0.3 s in centroid mode and in high energy. The transfer collision energy and trap collision energy were set at 30 V and 6 V for high energy acquisition respectively. The source parameters were set as follows: 2.5 kV for negative scan, sampling cone 25 V, extraction cone 3 V, source temperature 150uC, desolvation temperature 500 °C, desolvation gas flow 1000 L/h, and nebulizer gas 50 L/h. External calibration of the instrument was performed at the beginning of each batch of analysis by direct infusion of a sodium formate solution (10% formic acid/0.1 M NaOH/Acetonitrile at a ratio of 1/1/8) by controlling the mass accuracy from 40 to 2000 m/z (less than 5 ppm) and mass resolution (over 14,000, full width at half maximum, FWHM). LockMass calibration was applied using a solution of leucine enkephaline (0.5 mg/L, *m*/*z* 554.2620 for negative ion mode) at 0.1 mL/min.

### 3.6. NMR Analysis

The NMR measurements were performed with a Bruker-Avance 400 MHz spectrometer (Bruker BioSpin, Rheinstetten, Germany) operating with a stationary magnetic field of strength 9.4 T and equipped with a 5 mm BBI probe; the 90° proton pulse length was calibrated and established to be 9.4 µs, with a transmission power of 0 dB. The temperature was kept constant at 300.2 K. The residual protonated solvent was used as internal reference for the chemical shift scale (CHCl_3_, δ_1H_: 7.26 ppm, δ_13C_: 77.16 ppm).

The isolation of compounds present in fraction II was carried out on a Waters 2695 Alliance DAD System HPLC working in reversed phase with a Kinetex C18 column (100 Å pore size, 4.6 mm ID, 2.6 μm particle size, and 10 cm length, Phenomenex, Italy) using a mobile phase composed of solvent A, methanol:water (7:3 *v*/*v*) with 0.5% formic acid, and solvent B, methanol with 0.5% formic acid. The elution was performed in a gradient program starting with 70% B and reaching 100% B in 26 min. The isolated analytes ionised well in negative ion mode (S2).

After the preliminary survey, the pooled fractions III-IV were further purified by HPLC, and their main constituents’ structures were elucidated by NMR (using a combination of ^1^H, ^13^C, COSY, HMBC, and HSQC techniques).

### 3.7. Microorganisms and Media

For antifungal susceptibility testing, strains from the American Type Culture Collection (ATCC, Manassas, VA, USA; *S. cerevisiae* ATCC4040002 and *C. albicans* ATCC MYA-2876) were used as references. The environmental strain *S. cerevisiae* BB1533, and human gut clinical isolates *S.cerevisiae* YUC5, *C. albicans* YN5, *C. albicans* YN7, *C. albicans* YL1, *C. albicans* MFB 076N1, *C. albicans* MFB008MM1, *C. albicans* MFBYMS100-3, *C. albicans* MFBYMS102-2, *C. parapsilosis* YB1, *C. parapsilosis* MFBYMS100-1, *C. parapsilosis* MFB014CD7, *C. parapsilosis* MFB070N1, *C. glabrata* MFB004, *C. glabrata* MFB005FS4, *C. lusitaniae* MFB037-1, *C. lusitaniae* MFB022-2, *C. lusitaniae* MFB YMS100-16, *C. intermedia* MFB022-1, *C. intermedia* AD125, *C. tropicalis* MFB035-1, *C. tropicalis* RTT037-3, *C. pararugosa* MFB037N3, were kindly supplied by Professor Duccio Cavalieri Laboratory and tested for their susceptibility against the applied drugs.

Yeasts were grown on Sabouraud agarized medium for 48 h at 30 °C and resuspended in distilled water to a concentration of 1–5 × 10^5^ CFU/mL before testing.

### 3.8. Antifungal Susceptibility Testings

Yeasts were tested for their susceptibility to *H. mexicanum* crude extract (8 dilution series, ranging from 1000 μg/mL to 8 μg/mL) and fractionated extracts (7 dilution series ranging from 125 μg/mL to 2 μg/mL), following the European Committee for Antimicrobial Susceptibility Testing protocol (EUCAST Definitive Document EDef 7.2 Revision, 2012) [35]. Briefly, cells were grown in RPMI1640 medium supplied with 2% glucose, counted and inoculated at a concentration of 1–5 × 10^5^ CFU/mL. Minimum inhibitory concentration (MIC) values were determined using a spectrophotometer (at 530 nm) after 48 h and 120 h incubation, as the lowest concentration of the drug that resulted in a ≥50% inhibition of growth relative to the growth control [35].

### 3.9. Cytotoxicity Assay

The cytotoxicity of the isolated compounds was tested using the WST-8 (4-[3-(2-methoxy-4-nitrophenyl)-2-(4-nitrophenyl)-2*H*-5-tetrazolio]-1,3-benzene disulfonate sodium salt) conversion assay. The cytotoxicity was evaluated on two normal cell lines, namely PBMC and human foreskin fibroblast Hs27 (ATCC^®^ CRL-1634™, Manassas, VA, USA). PBMCs were obtained from buffy coat of healthy donors (as approved by the ethical committee of the local health center, Azienda Provinciale per i Servizi Sanitari, Provincia Autonoma di Trento) within 4 h from sample collection and isolated as previously described [9]. Cells were seeded in 96-well culture plates at concentration of 5000 cells/well and incubated for 24 h before treatment with *H. mexicanum* extracts and isolated compounds. To measure the cytotoxic effects 10 μL of Cell Counting Kit-8 (Sigma-Aldrich, St. Louis, MO, USA) (containing the WST-8 solution) were added to each well and incubated for 2h and 4h for fibroblasts and PBMCs respectively, at 37C before reading the absorbance at 450 nm.

The results were analyzed by Student’s *t*-test. The data are expressed as the percentage of viability and standard error of the mean.

### 3.10. Chemogenomics

Frozen aliquots of Yeast Deletion Heterozygous Diploid Pools (Cat. no.95401.H4Pool) were recovered for 10 generations and logarithmically growing cells were diluted to a final OD600 of 0,06 (=10^5^ cells/mL) in YPD containing 1% DMSO or compound. The compound was applied at a dose of 150 µg corresponding to 10–20% wild-type growth inhibition. Cells were grown in a Synergy 2 Multimode Microplate reader (BioTek, Winooski, VT, USA), harvested after 20 generations of growth and frozen at −20 °C for subsequent preparation of DNA. Genomic DNA was purified by Phenol:Chloroform:Isoamylalcohol extraction (including RNAse digestion). DNA quality was assessed via agarose gel electrophoresis and UV-Vis spectroscopy. For each condition (control or compound) 4 biological replicates were generated.

A two-step PCR protocol for efficient multiplexing of Bar-seq libraries was applied as previously described [36] with the following modifications. In a first step, UPTAGs and DNTAGs from a single sample were amplified using the primers Illumina UPTAG Index and Illumina UPkanMX and Illumina DNTAG Index and Illumina DNkanMX in separate PCR reactions. Illumina UPTAG and Illumina DNTAG primers contain a 5-bp sequence that uniquely identifies the sample. A complete list of primer sequences is provided in Supplementary Materials, Table S5.

Genomic DNA was normalized to 10 ng/mL and 100 ng were used as template for amplification of barcodes using FastStart High Fidelity PCR system (Roche Diagnostics), applying the following PCR program: 4 min at 95 °C followed by 30 cycles of 30 s at 95 °C, 30 s at 50 °C, 30 s at 72 °C, and a final extension step of 7 min at 72 °C. PCR products were confirmed on 2% agarose gels and purified using the Agencourt AMPure XP system (Beckman Coulter, Brea, CA, USA).

Purified PCR products were quantified using the Quant-iT dsDNA kit (Invitrogen) and 60 ng from each of the 4 different UPTAG libraries and, in a separate tube, 60 ng from each of the 4 different DNTAG libraries were combined. The multiplexed UPTAG libraries were then amplified using the primers P5 and Illumina UPkanMX, and the combined DNTAG libraries were amplified using the P5 and IlluminaDNkanMX primers (Table S5) and the PCR program: 4 min at 95 °C followed by 25 cycles of 10 sec at 95 °C, 10 sec at 50 °C, 10 sec at 72 °C, and a final extension step of 3 min at 72 °C. The 140-bp UPTAG and DNTAG libraries were purified using the Agencourt AMPure XP system, quantified using the Quant-iT dsDNA kit and combined in equimolar amounts. The library was sequenced on an Illumina HiSeq 2000 using standard methods, including the use of the standard Illumina sequencing primer (5′-ACA CTC TTT CCC TAC ACG ACG CTC TTC CGA TCT-3′). The qseq files for each of the 4 samples are available from the European Nucleotide Archive EBI-ENA with the accession number PRJEB27820.

### 3.11. Computational Details

For chemogenomic analysis read matching was performed using an in-house developed Python script which is available on Github (https://github.com/marcomoretto/Bar-seq). A Levenshtein distance of two was used to allow mismatches between reads. Reads perfectly matching multiple barcodes were removed from the analysis. The final count matrix has been obtained summing the UPTAG and DNTAG, removing barcodes with zeros across all samples and matches with low-count, since they are more likely due to sequencing noise.

The statistical analysis was performed with the voom transformation [37] package that estimates the mean–variance relationship of log counts, generating a precision weight for each observation that is fed into the limma empirical Bayes analysis pipeline [38]. A double threshold based on both *p*-value (≤0.01) and expression log2 fold change (≥15) was imposed to identify barcodes differentially abundant through pairwise comparison (Figure 5).

## 4. Conclusions

Novel dimeric acylphloroglucinols with potent selective cytotoxicity against pathogenic fungi have been isolated from a neglected plant species, highlighting the importance of conserving the biodiversity and investigation of the huge variety of plant derived structures for drug discovery. Genome-wide mechanism of action analysis allowed us to suggest novel potential drug targets for the development of antifungal therapies.

Taken together our results encourage further studies for the discovery and synthesis of dimeric acylphloroglucinol-based drugs as possible new leads in the antifungal pipeline.

## Figures and Tables

**Table 1:** Phenolic profile of extracts from *Hypericum mexicanum*.

Compound	Stem	Leaves Methanol Extracts *	Roots	Leaves Chloroform Extract *
Caffeic acid	nd	0.16 ± 0.10	nd	nd
3,5-dihydroxy benzoic acid	0.11 ± 0.05	2.02 ± 0.30	0.11 ± 0.05	0.13 ± 0.03
Gallic acid	nd	nd	nd	nd
Kaempferol	nd	1.78 ± 0.09	0.01 ± 0.01	0.40 ± 0.07
Quercetin-3-glucuronide	nd	0.09 ± 0.01	nd	0.21 ± 0.04
Quercetin	nd	97.07 ± 1.01	0.06 ± 0.01	2.87 ± 0.60
Quercetin-3-glucoside	0.05 ± 0.01	86.46 ± 3.03	0.04 ± 0.01	nd
Kaempferol-3-glucuronide	nd	nd	nd	0.01 ± 0.01
Isorhamnetin	0.02 ± 0.01	6.73 ± 0.02	nd	0.03 ± 0.01
Syringetin-3-glucoside	nd	nd	nd	nd
Isorhamnetin-3-glucoside	0.12 ± 0.01	96.58 ± 5.02	nd	0.13 ± 0.03
Myricetin	nd	1.80 ± 0.10	nd	nd
Catechin	nd	nd	nd	nd
Epicatechin	0.09 ± 0.03	36.17 ± 2.01	nd	nd
Gallocatechin	nd	0.12 ± 0.05	nd	nd
Epigallocatechin	nd	nd	nd	nd
Procyanidin B1	nd	2.64 ± 0.30	nd	0.09 ± 0.03
Procyanidin B2	0.24 ± 0.03	117.37 ± 4.02	2.72 ± 0.31	0.19 ± 0.08
*cis*-piceid	nd	0.11 ± 0.06	nd	nd
Vanillic acid	nd	0.47 ± 0.12	0.01 ± 0.01	0.08 ± 0.01
Esculin	nd	0.04 ± 0.01	nd	0.01 ± 0.01
Neochlorogenic acid	nd	6.98 ± 0.50	0.01 ± 0.01	nd
Cryptochlorogenic acid	nd	0.36 ± 0.02	nd	nd
Chlorogenic acid	nd	nd	nd	nd
Luteolin	nd	2.81 ± 0.81	nd	0.44 ± 0.09
Luteolin-7- glucoside	nd	0.42 ± 0.05	nd	traces
Quercetin-3-arabinoside	nd	2.39 ± 0.81	nd	nd
2,6-dihydroxy benzoic acid	nd	nd	nd	nd
*p*-hydroxybenzoic acid	nd	1.31 ± 0.31	0.02 ± 0.01	0.04 ± 0.01
Cinnamic acid	nd	0.08 ± 0.01	nd	0.13 ± 0.01
Naringenin-7-glucoside	nd	0.12 ± 0.07	nd	1.74 ± 0.30
Coniferyl alcohol	nd	nd	nd	nd
Kaempferol-3-glucoside	0.01 ± 0.01	5.40 ± 1.01	0.01 ± 0.01	nd
Phlorizin	0.02 ± 0.01	4.51 ± 0.51	0.03 ± 0.01	nd

* Data expressed as µg/g extract.

**Table 2:** Anti-*Candida* activity of leaves extracts from *H. mexicanum*.

Strain (N° Tested)	Chloroformic	Methanol
		MIC_50_ µg/mL *
*C. albicans* (4)	27	64
*C. parapsilosis* (2)	16	125
*C. glabrata* (2)	11	45
*C. lusitaniae* (2)	10	32
*C. tropicalis* (1)	23	>125
*C. pararugosa* (1)	32	>125
*C. deformans* (1)	32	>125

* Data are expressed as geometric mean.

**Table 3:** Anti-*Candida* activity of fractions collected after chromatographic separation of *H. mexicanum* lipophilic extract.

Strain	Fraction I	Fraction II	Fluconazole
	MIC_50_ (µg/mL)		
*C. albicans* MFB 051-1	>50	<32	64.0 ± 1.0
*C. albicans* MFB032-1	>50	32 ± 1.0	0.5 ± 0.1
*C. albicans* MFB076-1	>50	32 ± 0.5	>64
*C. albicans* MFB008 MM1	>50	<4	>64
*C. albicans* YMS 102-2	>50	<<4	>64
*C. albicans* YMS100-3	>50	16 ± 0.5	0.5 ± 0.1
*C. lusitaniae* MFB037 N1	>50	8 ± 1.0	0.5 ± 0.2
*C. lusitaniae* YMS 100-16	>50	<4	0.3 ± 0.1
*C. parapsilosis* MFB014 CD7	>50	4 ± 0.5	>64
*C. parapsilosis* MFB070 N1	>50	125 ± 1	>64
*C. parapsilosis* YMS 100-1	>50	4 ± 0.5	2 ± 0.5
*C. pararugosa* MFB037 N3	>50	8 ± 0.5	0.1

MIC_50_ values are expressed as means ± standard deviation of three independent experiments.

**Table 4:** NMR spectroscopic data for compounds **2** and **5** (400 MHz, CDCl_3_, 300K).

Signal	^1^ H-NMR Compound 2	^1^ H-NMR Compound 5	^13^ C-NMR Compound 2	^13^ C-NMR Compound 5
1			199.4 s	199.4 s
2			107.1 s	107.1 s
3			171.6 s	171.6 s
4			44.3 s	44.3 s
5			187.3 s	187.3 s
6			111.2 s	111.4 s
7	3.55 (2H; br d; 16.9)	3.54 (2H; br d; 16.9)	16.9 br t	17.0 t
8	1.45 (3H; s)	1.45 (3H; s)	24.2 brq	19.1 brq
9	1.53 (3H; s)	1.53 (3H; s)	25.4 brq	19.5 brq
10	4.21 (1H; septet; 6.8)	4.21 (1H; septet; 6.8)	210.9 s	210.8 s
11			36.6 d	36.6 d
12	1.18 (3H, s)	1.18 (3H, s)	19.3 brq	19.3 brq
13	1.18 (3H, s)	1.18 (3H, s)	16.7 brq	20.5 brq
2′			78.1 s	79.5 s
2′-Me	1.48 s	1.47 s	27.7 brq	27.5 brq
	1.48 s	1.23	28.1 brq	20.5 brq
3′	5.44 (d, 10.1)	1.71 (1H, m)	124.6 d	40.8 d
4′	6.70 (d,10.1)	2.83 (1H; dd,5.4,17.1)	117.3 d	22.7 t
		2.17 (1H; dd 10.2,17.1)		
5′			155.4 s	155.3 s
6′			108.1 s	107.1 s
7′			162.2 s	161.6 s
8′			104.2 s	104.7 s
9′			159.3 s	161.4 s
10′			103.6 s	102.4 s
11′			210.8 s	210.7 s
12′	3.81(1H; septet; 6.8)	3.89(1H; septet; 6.8)	45.7 d	38.9 d
13′	1.18 (3H; s)	1.18 (3H; s)	19.1 q	19.1 q
14′	1.42/1.88 (2H; m)	1.42/1.88 (2H; m)	26.7 t	27.5 t
15′	0.94 (3H; t; 6.7)	0.94 (3H; t; 6.7)	11.9 q	11.9 q
1′’		2.32/1.81 (2H; m)		29.4 t
2′’		5.19 (1H, br t, 6.8)		122.1 d
3′’				133.1 s
4′’		1.73 (3H; brs)		25.8 q
5′’		1.62 (3H. brs)		17.2 q

**Table 5:** High resolution MS data for parent and daughter ions of acylphloroglucinols **2**–**6** as measured by HR-ESI-QTOFMS.

Compound	ESI (−) Ion Type	Molecular Formula	Calculated Exact Mass (*m*/*z*)	High Resolution Mass Measurement (±0.0010)
**2**	[M − H]^−^	C_29_H_35_O_8_	511.2337	511.2315
	Fragment-ion C2-C7	C_12_H_15_O_4_	223.0976	223.0978
	Fragment-ion C6′-C7	C_13_H_15_O_4_	235.0976	235.0978
	Fragment-ion C6′-C7	C_16_H_19_O_4_	275.1289	
**3**	[M − H]^−^	C_30_H_37_O_8_	525.2493	525.2483
	Fragment-ion C2-C7	C_13_H_17_O_4_	237.1132	237.1133
	Fragment-ion C6′-C7	C_14_H_17_O_4_	249.1132	
	Fragment-ion C6′-C7	C_16_H_19_O_4_	275.1289	275.1293
**4**	[M − H]^−^	C_33_H_43_O_8_	567.2943	567.2941
	Fragment-ion C2-C7	C_12_H_15_O_4_	223.0976	223.0978
	Fragment-ion C6′-C7	C_13_H_15_O_4_	235.0976	235.0978
	Fragment-ion C6′-C7	C_20_H_27_O_4_	331.1915	331.1918
**5**	[M − H]^−^	C_34_H_45_O_8_	581.3120	581.3111
	Fragment-ion C2-C7	C_12_H_15_O_4_	223.0976	223.0978
	Fragment-ion C6′-C7	C_13_H_15_O_4_	235.0976	235.0978
	Fragment-ion C6′-C7	C_21_H_29_O_4_	345.2071	
**6**	[M − H]^−^	C_35_H_47_O_8_	595.3276	595.3267
	Fragment-ion C2-C7	C_13_H_17_O_4_	237.1132	237.1141
	Fragment-ion C6′-C7	C_14_H_17_O_4_	249.1132	249.1120
	Fragment-ion C6′-C7	C_21_H_29_O_4_	345.2071	345.2075

**Table 6:** Anti-*Candida* activity of two major isolated compounds.

*Candida* Strain	Uliginosin C	3′ Prenyl Uliginosin B	Fluconazole
	MIC_50_ (µM)		
*C. albicans* MFB 076N1	16 ± 0.5	15 ± 1	208 ± 2
*C. albicans* MFB 008 MM1	6 ± 0.2	3 ± 0.2	208 ± 2
*C. albicans* MFB YMS 100-3	>32	>30	1.6 ± 0.1
*C. albicans* MFB YMS 102-2	8 ± 0.7	4 ± 0.3	>208
*C. parapsilosis* MFB YMS 100-1	32 ± 1	6 ± 0.6	6 ± 0.5
*C. parapsilosis* MFB 014 CD7	32 ± 1	30 ± 2	>208
*C. parapsilosis* MFB 070 N1	>32	>30	>208
*C. lusitaniae* MFB YMS 100-16	>32	30 ± 2	0.8 ± 0.2
*C. lusitaniae* MFB 037 N1	8 ± 0.2	30 ± 1	1.6 ± 0.5
*C. pararugosa* MFB 037 N3	8 ± 0.7	15 ± 2	0.4 ± 0.0
*C. glabrata* MFB004	16 ± 1	4 ± 0.1	0.13 ± 0.0
*C. glabrata* MFB005FS4	8 ± 0.4	6 ± 0.1	0.13 ± 0.0

MIC_50_ values are expressed as means ± standard deviation of three independent experiments.

## Supplementary Materials

The following are available online at https://www.mdpi.com/2218-1989/10/11/459/s1, Figure S1: Characterization of Fraction I, Figure S2: Characterization of Fraction II, Table S1: Antifungal activity of *H. mexicanum* crude extract, Table S2: Anti-Candida activity of *H. mexicanum* extracts, Table S3: Antifungal activity of isolated compounds, Table S4: Top twenty sensitive and resistant deletion mutants, Table S5: Complete list of primer sequences.
