# Supplementary material for: Potent Antifungal Properties of Dimeric Acylphloroglucinols from Hypericum mexicanum and Mechanism of Action of a Highly Active 3′Prenyl Uliginosin B
Source: Metabolites. 2020 Nov 13;10(11):459. doi: 10.3390/metabo10110459 (PMC7697946; doi:10.3390/metabo10110459)
Supplement: Supplementary file 1 [file metabolites-10-00459-s001.zip › Supplementary Figures.pdf]

Figure S1: Characterization of Fraction I. **A)** HPLC-MS analysis: the extracted chromatogram (λ 240nm) of Fraction I shows that it is mainly composed of a single species. **B)** HPLC-UV analysis: UV spectrum of the main compound of Fraction I

A)

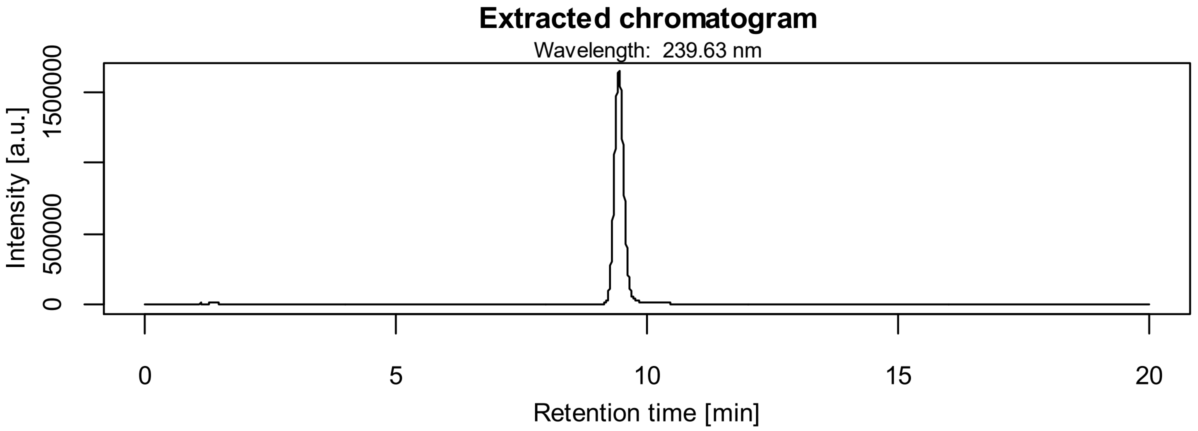

B)

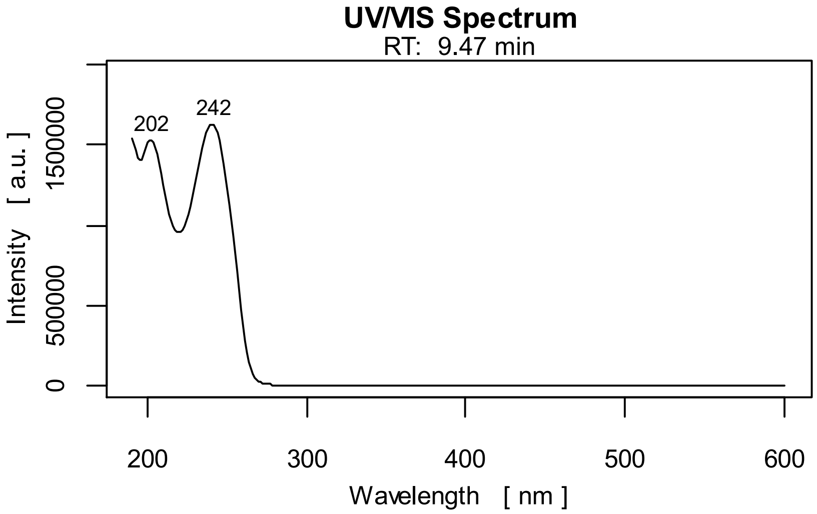

Figure S2: Characterization of Fraction II A) HPLC-MS analysis: the extracted chromatogram (λ 280nm) of Fraction III-IV. **B)** HPLC-UV analysis: UV spectra of the main constituents of Fraction II.

A)

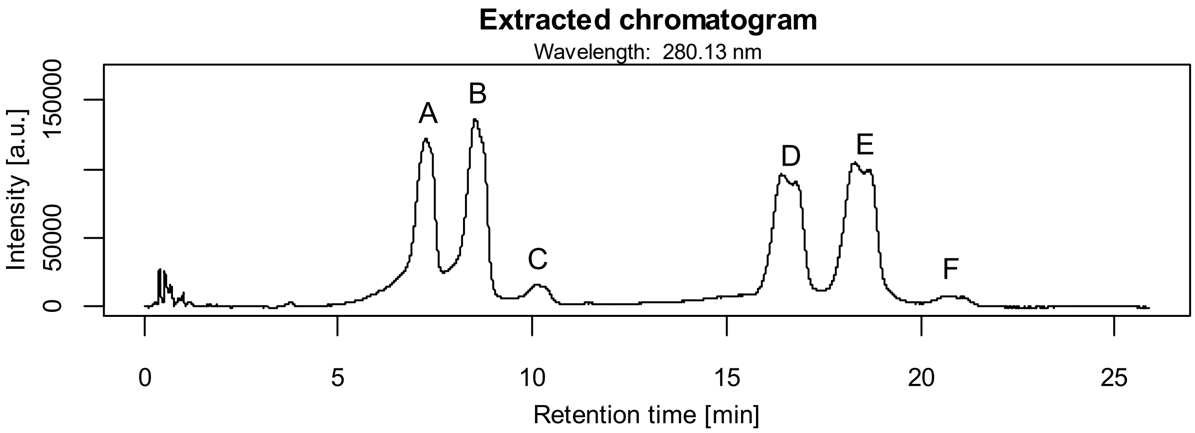

B)

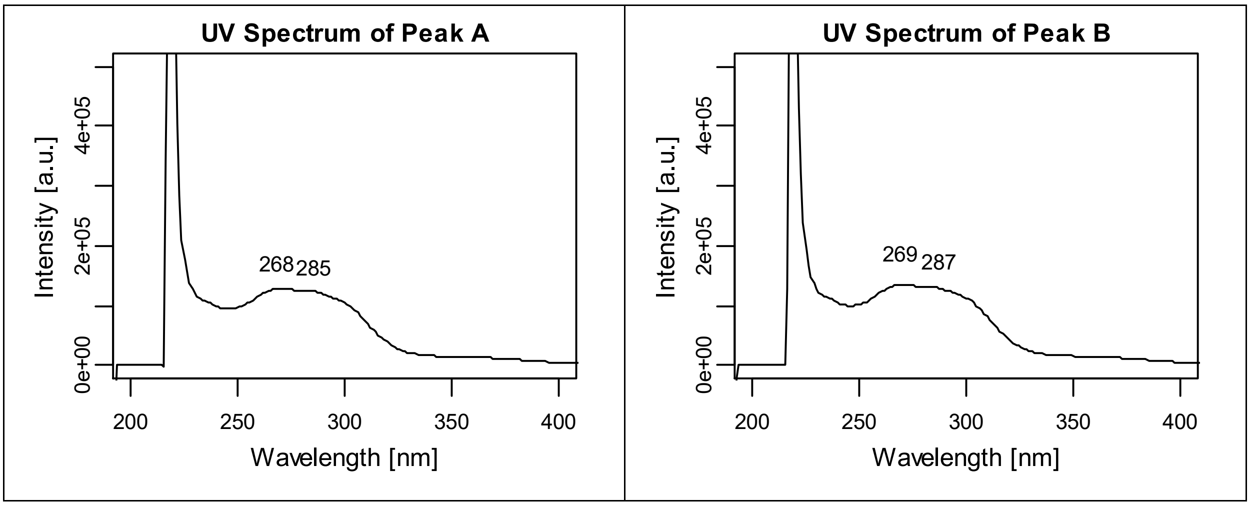

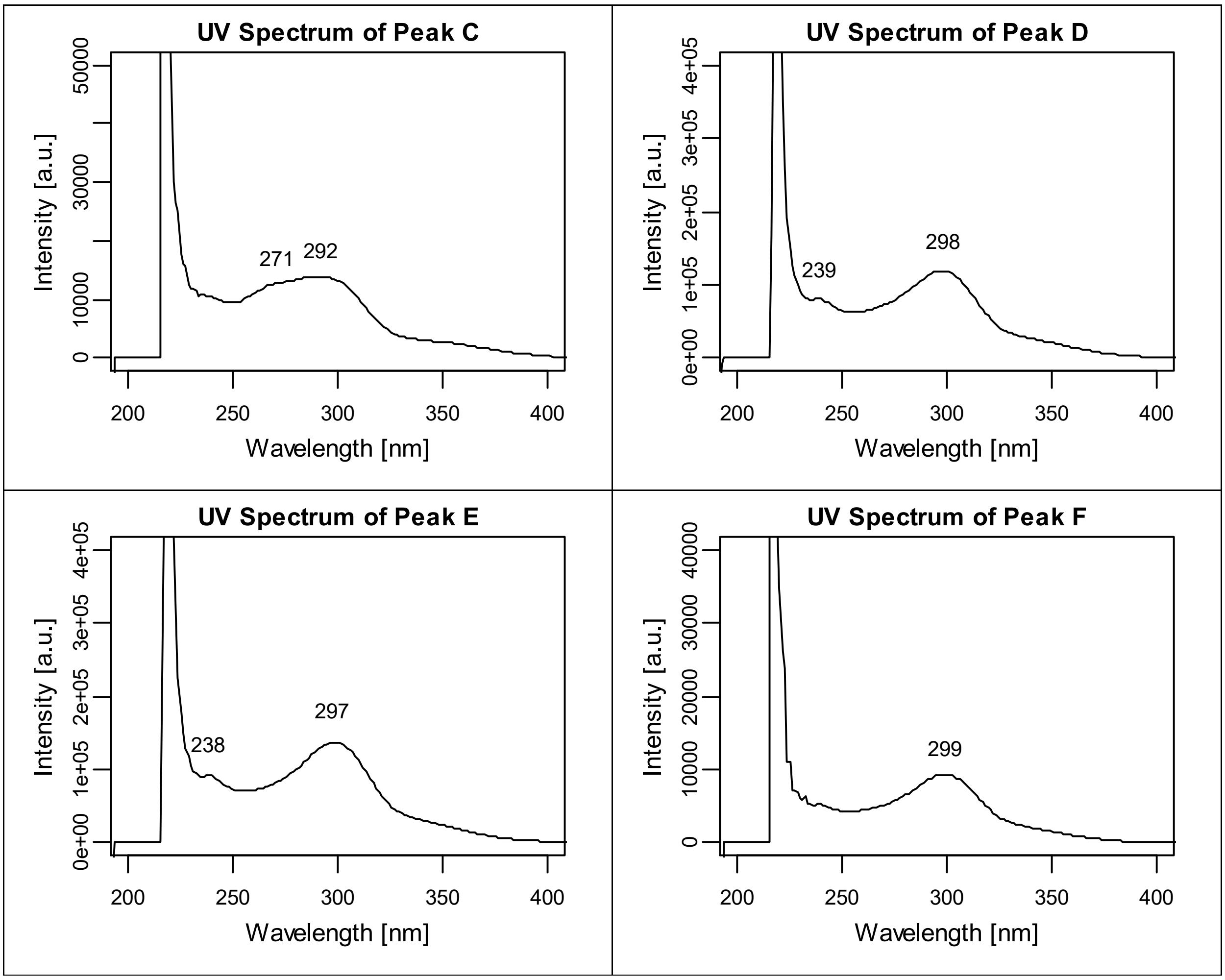
UV Spectrum of Peak C
271
292
UV Spectrum of Peak D
239
298
UV Spectrum of Peak E
238
297
UV Spectrum of Peak F
299
Intensity [a.u.]
Wavelength [nm]
