# Supplementary material for: Potent Antifungal Properties of Dimeric Acylphloroglucinols from Hypericum mexicanum and Mechanism of Action of a Highly Active 3′Prenyl Uliginosin B
Source: Metabolites. 2020 Nov 13;10(11):459. doi: 10.3390/metabo10110459 (PMC7697946; doi:10.3390/metabo10110459)
Supplement: Supplementary file 1 [file metabolites-10-00459-s001.zip › Supplementary Tables.pdf]

Table S1: Aantifungal activity of *H. mexicanum* crude extract

**Fungal strain**	**MIC$_{50}$ (µg/ml)***	**MIC$_{50}$ mean (µg/ml)****
S. cerevisiae SGU421	16 ± 0,03	44,72
S. cerevisiae BB1533	125 ± 0,16	
C. albicans YN5	236± 0,21	63,5
C. albicans YN7	64± 0,04	
C. albicans YL1	32± 0,01	

*Data are expressed as the mean ± standad deviation of three independent measurements
**Data are expressed as geometric mean

Table S2: Anti-*Candida* activity of *H. mexicanum* extracts

Strain	Chloroform	Methanol
C. albicans YN5	32	64
C. albicans YN7	32	64
C. albicans YD7	32	<125
C. albicans YD8	16	<125
C. parapsilosis YV1	>16	<125
C. parapsilosis YV2	32	<125
C. glabrata MFB 005-4	>16	16
C. glabrata MFB 004-1	16	125
C. lusitaniae MFB0037-1	16	32
C. lusitanie MFB 0022-1	>16	32
C. tropicalis MFB0035-1	16	<125
C. pararugosa MFB 007-3	32	<125
C. deformans MFB 037-1	32	<125

*Data are expressed as the mean ± standad deviation of three independent measurements

Table S3: Antifungal activity of isolated compounds

Strain	**Compound 1 MIC$_{50}$ (µM)**	**Compound 2**	**Compound 3**	**Compound 4**	**Compound 5**
C. albicans MFB008 MM1	6±0.23	8±0.91	4±0.32	3±0.16	3±0.14
C. albicans YMS 102-2	8±0.71	8±0.93	4±0.35	4±0.28	4±0.23
C. glabrata MFB004	16± 1.14	32±1.73	6±0.23	4±0.11	3±0.19
C. glabrata MFB005FS4	8±0.43	8±1.26	10±0.82	6±0.13	3±0.14

MIC$_{50}$ values are expressed as means ± standard deviation of three independent experiments.

Table S4: Top twenty sensitive and resistant deletion mutants

GENES	LogFC	PValue	CHR	DESCRIPTION
**Sensitive Mutants**				
CCT5	-3.4	0.003	10	Subunit of the cytosolic chaperonin Cct ring complex; related to Tcp1p, required for the assembly of actin and tubulins in vivo
YPR053C	-3.2	0.008	16	Putative protein of unknown function; conserved among S. cerevisiae strains; YPR053C is not an essential gene; partially overlaps verified ORF NHP6A/YPR052C
YKR040C	-3.1	0.001	11	Dubious open reading frame; unlikely to encode a functional protein, based on available experimental and comparative sequence data; partially overlaps the uncharacterized ORF YKR041W
PDX3	-3.1	0.004	2	Pyridoxine (pyridoxamine) phosphate oxidase; has homologs in E. coli and Myxococcus xanthus; transcription is under the general control of nitrogen metabolism
TDA9	-3.0	0.027	13	Transcription factor that regulates acetate production; green fluorescent protein (GFP)-fusion protein localizes to the nucleus; null mutant is sensitive to expression of the top1-T722A allele; not an essential gene; TDA9 has a paralog, RSF2, that arose from the whole genome duplication
YPR096C	-3.0	0.016	16	Protein of unknown function; may interact with ribosomes, based on co-purification experiments
TSL1	-3.0	0.016	13	Large subunit of trehalose 6-phosphate synthase/phosphatase complex; Tps1p-Tps2p complex converts uridine-5'-diphosphoglucose and glucose 6-phosphate to trehalose; contributes to survival to acute lethal heat stress; mutant has aneuploidy tolerance; protein abundance increases in response to DNA replication stress; TSL1 has a paralog, TPS3, that arose from the whole genome duplication
SCT1	-3.0	0.016	2	Glycerol 3-phosphate/dihydroxyacetone phosphate sn-1 acyltransferase; dual substrate-specific acyltransferase of the glycerolipid biosynthesis pathway; prefers 16-carbon fatty acids; similar to Gpt2p; gene is constitutively transcribed
MTC7	-2.9	0.006	5	Protein of unknown function; predicted metabolic role based on network analysis derived from ChIP experiments, a large-scale deletion study and localization of transcription factor binding sites; null mutant is sensitive to temperature oscillation in a cdc13-1 mutant
GSP1	-2.8	0.002	12	Ran GTPase; GTP binding protein (mammalian Ranp homolog) involved in the maintenance of nuclear organization, RNA processing and transport; regulated by Srm1p, Rna1p, Yrb1p, Yrb2p, Yrp4p, Yrb30p, Cse1p and Kap95p; GSP1 has a paralog, GSP2, that arose from the whole genome duplication
GGA1	-2.7	0.010	4	Golgi-localized protein with homology to gamma-adaptin; interacts with and regulates Arf1p and Arf2p in a GTP-dependent manner in order to facilitate traffic through the late Golgi; GGA1 has a paralog, GGA2, that arose from the whole genome duplication
RPL41B	-2.7	0.011	4	Ribosomal 60S subunit protein L41B; comprises only 25 amino acids; rpl41a rpl41b double null mutant is viable; homologous to mammalian ribosomal protein L41, no bacterial homolog; RPL41B has a paralog, RPL41A, that arose from the whole genome duplication
FAR10	-2.7	0.083	12	Protein involved in recovery from arrest in response to pheromone; acts in a cell cycle arrest recovery pathway independent from Far1p; interacts with Far3p, Far7p, Far8p, Far9p, and Far11p; potential Cdc28p substrate; FAR10 has a paralog, VPS64, that arose from the whole genome duplication

HUL4	-2.7	0.015	10	Protein with similarity to hect domain E3 ubiquitin-protein ligases; not essential for viability; found in association with Trf4 in TRAMP complex
TLG1	-2.6	0.032	4	Essential t-SNARE that mediates fusion of vesicles with the late Golgi; forms a complex with Tlg2p and Vti1p; mediates fusion of endosome-derived vesicles with the late Golgi; binds the docking complex VFT (Vps fifty-three) through interaction with Vps51p
MAP1	-2.6	0.000	12	Methionine aminopeptidase; catalyzes the cotranslational removal of N-terminal methionine from nascent polypeptides; function is partially redundant with that of Map2p
ERG29	-2.5	0.003	13	Protein of unknown function involved in ergosterol biosynthesis; conditional mutants produce less ergosterol, display impaired oxygen consumption, respiratory growth, mitochondrial iron utilization, and are more sensitive to oxidative stress; mutant bm-8 has a growth defect on iron-limited medium that is complemented by overexpression of Yfh1p; protein localizes to the cytoplasm, ER and nuclear envelope; highly conserved in ascomycetes
SNT1	-2.5	0.082	3	Subunit of the Set3C deacetylase complex; interacts directly with the Set3C subunit, Sif2p; putative DNA-binding protein; mutant has increased aneuploidy tolerance; relocalizes to the cytosol in response to hypoxia
NPL4	-2.5	0.065	2	Substrate-recruiting cofactor of the Cdc48p-Npl4p-Ufd1p segregase; assists Cdc48p in the dislocation of misfolded, polyubiquitinated ERAD substrates that are subsequently delivered to the proteasome for degradation; also involved in the regulated destruction of resident ER membrane proteins, such as HMG-CoA reductase (Hmg1/2p) and cytoplasmic proteins (Fbp1p); role in mobilizing membrane bound transcription factors by regulated ubiquitin/proteasome-dependent processing (RUP)
AKR1	-2.5	0.019	4	Palmitoyl transferase involved in protein palmitoylation; acts as a negative regulator of pheromone response pathway; required for endocytosis of pheromone receptors; involved in cell shape control; contains ankyrin repeats; AKR1 has a paralog, AKR2, that arose from the whole genome duplication; any of several human homologs encoding DHHC-type zinc fingers (ZDHHC) can complement temperature sensitivity of yeast akr1 null mutant
**Resistant Mutants**				
IOC2	4.1	0.001	12	Subunit of the Isw1b complex; exhibits nucleosome-stimulated ATPase activity and acts within coding regions to coordinate transcription elongation with termination and processing; contains a PHD finger motif; other complex members are Isw1p and Ioc4p
NOP6	3.5	0.033	4	rRNA-binding protein required for 40S ribosomal subunit biogenesis; contains an RNA recognition motif (RRM); hydrophilin essential to overcome the stress of the desiccation-rehydration process; NOP6 may be a fungal-specific gene as no homologs have been yet identified in higher eukaryotes
PMP1	3.3	0.044	3	Regulatory subunit for the plasma membrane H(+)-ATPase Pma1p; small single-membrane span proteolipid; forms unique helix and positively charged cytoplasmic domain that is able to specifically segregate phosphatidylserines; PMP1 has a paralog, PMP2, that arose from the whole genome duplication
RPC10	3.2	0.002	8	RNA polymerase subunit ABC10-alpha, found in RNA pol I, II, and III; relocalizes from nucleolus to cytoplasm upon DNA replication stress
RMD5	3.1	0.030	4	Component of GID Complex that confers ubiquitin ligase (U3) activity; necessary for polyubiquitination and degradation of the gluconeogenic enzyme fructose-1,6-bisphosphatase; forms dimer with Fyv10p that is then

				recruited to GID Complex by Gid8p; also required for sporulation; conserved protein that has a degenerate RING finger domain
SSH1	3.0	0.012	2	Subunit of the Ssh1 translocon complex; Sec61p homolog involved in co-translational pathway of protein translocation; not essential
CTA1	3.0	0.012	4	Catalase A; breaks down hydrogen peroxide in the peroxisomal matrix formed by acyl-CoA oxidase (Pox1p) during fatty acid beta-oxidation
YML079W	2.9	0.026	13	Non-essential protein of unknown function; has structural resemblance to plant storage and ligand binding proteins (canavalin, glycinin, auxin binding protein) and to some enzymes (epimerase, germin); localizes to the nucleus and cytoplasm
THG1	2.9	0.006	7	tRNAHis guanylyltransferase; adds a guanosine residue to the 5' end of tRNAH is after transcription and RNase P cleavage; can also catalyze reverse (3'-5') polymerization with certain substrates in a template-dependent reaction; couples nuclear division and migration to cell budding and cytokinesis; essential enzyme conserved among eukaryotes
MUB1	2.7	0.020	13	MYND domain-containing protein; component of the Mub1p-Ubr2p-Rad6p ubiquitin ligase complex, required for ubiquitination and degradation of Rpn4p; interacts with Ubr2p (E3) and indirectly with Rad6p (E2); short-lived protein degraded in a Ubr2p/Rad6p dependent manner; proposed to function as both a partner and substrate of the Ubr2p/Rad6p ubiquitin ligase; similar to the A. nidulans samB gene
RCF1	2.6	0.070	13	Cytochrome c oxidase subunit; required for assembly of the Complex III-Complex IV supercomplex, and for assembly of Cox13p and Rcf2p into cytochrome c oxidase; similar to Rcf2p, and either Rcf1p or Rcf2p is required for late-stage assembly of the Cox12p and Cox13p subunits and for cytochrome c oxidase activity; required for growth under hypoxic conditions; member of the hypoxia induced gene family; C. elegans and human orthologs are functional in yeast
YDL206W	2.6	0.040	4	Putative protein of unknown function; YDL206W is not an essential protein
YJR087W	2.5	0.005	10	Dubious open reading frame; unlikely to encode a functional protein, based on available experimental and comparative sequence data; not conserved in closely related Saccharomyces species; partially overlaps the verified genes STE18 and ECM2
IZH1	2.5	0.047	4	Membrane protein involved in zinc ion homeostasis; member of the four-protein IZH family; transcription is regulated directly by Zap1p, expression induced by zinc deficiency and fatty acids; deletion increases sensitivity to elevated zinc; IZH1 has a paralog, IZH4, that arose from the whole genome duplication
DUS1	2.4	0.029	13	Dihydrouridine synthase; member of a widespread family of conserved proteins including Smm1p, Dus3p, and Dus4p; modifies pre-tRNA(Phe) at U17
POR2	2.4	0.002	9	Putative mitochondrial porin (voltage-dependent anion channel); not required for mitochondrial membrane permeability or mitochondrial osmotic stability; POR2 has a paralog, POR1, that arose from the whole genome duplication
ABM1	2.3	0.009	10	Protein of unknown function; required for normal microtubule organization
YNL011C	2.2	0.007	14	Putative protein of unknown function; YNL011C is not an essential gene
YNL095C	2.2	0.005	14	Putative protein of unknown function; predicted to contain a transmembrane domain; not an essential gene; YNL095C has a paralog, ECM3, that arose from the whole genome duplication

NEL1	2.2	0.277	8	Activator of Sar1p GTPase activity; paralog of Sec23 but does not associate with the COPII components; not an essential gene

Table S5. Complete list of primer sequences

**oligoname**	**Sequence 5′ - 3′**
UPTAG Index 1	ACG CTC TTC CGA TCT ATACC GTC CAC GAG GTC TCT
UPTAG Index 2	ACG CTC TTC CGA TCT TCTAG GTC CAC GAG GTC TCT
IUPTAG Index 3	ACG CTC TTC CGA TCT GCAGC GTC CAC GAG GTC TCT
UPTAG Index 4	ACG CTC TTC CGA TCT CCGAG GTC CAC GAG GTC TCT
DNTAG Index 1	ACG CTC TTC CGA TCT ATACC GTG TCG GTC TCG TAG
DNTAG Index 2	ACG CTC TTC CGA TCT TCTAG GTG TCG GTC TCG TAG
DNTAG Index 3	ACG CTC TTC CGA TCT GCAGC GTG TCG GTC TCG TAG
DNTAG Index 4	ACG CTC TTC CGA TCT CCGAG GTG TCG GTC TCG TAG
UPTAG_Index_5	ACG CTC TTC CGA TCT AAGAT GTC CAC GAG GTC TCT
UPTAG_Index_6	ACG CTC TTC CGA TCT TAGTA GTC CAC GAG GTC TCT
UPTAG_Index_7	ACG CTC TTC CGA TCT GAACT GTC CAC GAG GTC TCT
UPTAG_Index_8	ACG CTC TTC CGA TCT CAATG GTC CAC GAG GTC TCT
DNTAG_Index_5	ACG CTC TTC CGA TCT AAGAT GTG TCG GTC TCG TAG
DNTAG_Index_6	ACG CTC TTC CGA TCT TAGTA GTG TCG GTC TCG TAG
DNTAG_Index_7	ACG CTC TTC CGA TCT GAACT GTG TCG GTC TCG TAG
DNTAG_Index_8	ACG CTC TTC CGA TCT CAATG GTG TCG GTC TCG TAG
UPkanMX	CAA GCA GAA GAC GGC ATA CGA GAT GTC GAC CTG CAG CGT ACG
DNkanMX	CAA GCA GAA GAC GGC ATA CGA GAT ACG AGC TCG AAT TCA TCG
P5	A ATG ATA CGG CGA CCA CCG AGA TCT ACA CTC TTT CCC TAC ACG ACG CTC TTC CGA TCT
